# Rhizophora mucronata var. alokii – a new variety of mangrove species from the Andaman and Nicobar Islands, India (Rhizophoraceae)

**DOI:** 10.3897/phytokeys.52.5037

**Published:** 2015-07-13

**Authors:** P. Ragavan, P. M. Mohan, R. S. C. Jayaraj, K. Ravichandran, S. Saravanan

**Affiliations:** 1 Department of Ocean studies and Marine Biology, Pondicherry University, Brookshabad Campus, Port Blair, Andaman & Nicobar Islands, India; 2 Department of Environment and Forests, Arunachal Pradesh, India; 3 Department of Environment and Forests, Andaman and Nicobar Administration, Port Blair, A & N Islands, India; 4 Institute of Forest Genetics and Tree Breeding, Coimbatore, Tamil Nadu, India

**Keywords:** Rhizophoraceae, Rhizophora mucronata var. alokii, new variety, Andaman and Nicobar Islands, India

## Abstract

Rhizophora mucronata var. alokii (Rhizophoraceae), a new variety of *Rhizophora* from the Andaman and Nicobar Islands, India, is described and illustrated. The new variety is remarkable in having four stamens, laterally folded leaves, a short peduncle, thick leathery petals, and a four-sided ovary with a sessile style. A key for the species of *Rhizophora* of the Andaman and Nicobar Islands is also provided.

## Introduction

The genus *Rhizophora* is the most common mangrove genus worldwide. Two species (*Rhizophora mangle* L. and *Rhizophora racemosa* G. Mey) and one natural hybrid (Rhizophora × harrisonii Leechm.) are restricted to the Atlantic-East Pacific Region, three species (*Rhizophora apiculata* Blume, *Rhizophora mucronata* Lam., and *Rhizophora stylosa* Griff.) and four named natural hybrids (Rhizophora × annamalayana Kathiresan, Rhizophora × lamarckii Montrouz, Rhizophora × selala (Salvoza) Toml., and Rhizophora × tomlinsonii Duke) are restricted to the Indo-West Pacific (IWP) region, and one species (*Rhizophora samoensis* (Hochr.) Salvosal) extends into both regions (Duke and Bunt 1979, Duke 1992, Duke et al. 1998, Duke 2002, Duke 2010). In addition, Ng et al. (2013) recognized an unnamed hybrid between *Rhizophora mucronata* and *Rhizophora stylosa* through molecular studies. All the IWP taxa except *Rhizophora samoensis* and Rhizophora × selala are known from India (Ragavan et al. 2011).

The mangroves of the Andaman and Nicobar Islands (ANI) are denser and more diverse compared to other mangrove habitats in India (Mandal and Naskar 2008). According to the latest estimate by the Forest Survey of India (FSI 2013), the total mangrove area is approximately 4,628 km^2^ in India, of which 604 km^2^ occurs in the ANI. A total of 38 mangrove species has been recorded from the ANI. These include five *Rhizophora* taxa (*Rhizophora apiculata*, *Rhizophora mucronata*, *Rhizophora stylosa*, Rhizophora × lamarckii and Rhizophora × annamalayana; Ragavan et al. 2011).

During a recent botanical excursion, an interesting population of *Rhizophora* was encountered in the mangrove forest of Austin Creek, North Andaman. Specimens were collected and did not match any of the known species of the genus and hence have been described and illustrated here as new.

## Materials and methods

To better assess the taxonomic placement and distinguishing characteristics of the new taxon, a morphometric analysis of the *Rhizophora* taxa present in ANI was performed. Seventeen attributes of leaves, inflorescences and flowers (Table 1) were examined for each taxon. The dataset was used for cluster analysis with Primer-e software (Version 6). Results of the cluster analysis were then used to select the taxon morphologically most similar to the new entity. T-tests were used to determine which attributes differed significantly between the two taxa. A key for the *Rhizophora* species of Andaman and Nicobar Islands has also been provided to facilitate identification.

**Table 1.** Characters used for classification analysis of *Rhizophora* species of the ANI; average value (range) in cm for each taxon. Where no range is included, the values for the taxon showed no variation. Values that differ significantly (p < 0.05) between the varieties of R. mucronata are marked with an asterisk.

Characters	*Rhizophora apiculata*	Rhizophora mucronata var. mucronata	Rhizophora mucronata var. alokii	*Rhizophora stylosa*	Rhizophora × annamalayana	Rhizophora × lamarckii
Leaf length	13 (8.5–16.2)	13.55* (8.5–18)	11.29* (7–13)	11.1 (8–13)	12.39 (10–16)	13.08 (8–16)
Leaf width	5.9 (4–8.5)	8.47 (5.7–11.3)	6.71 (4–8.5)	5.68 (4–6.3)	7.4 (6–12)	6.45 (4.5–8.5)
Leaf length width ratio	2 (1.7–3.12)	1.6* (1.4–1.8)	1.69* (1.43–1.79)	2.02 (1.8–2.8)	1.67 1.4–1.7	2 (1.79–2.2)
Leaf mucro length	0.4 (0.4–0.5)	0.5 (0.4–0.6)	0.45 (0.4–0.5)	0.5 (0.4–0.6)	0.34 (0.3–0.5)	0.45 (0.4–0.5)
Petiole length	1.8 (1.4–2.5)	2.61* (1.5–3)	2.22* (1.5–3)	3.35 (2–3.5)	2.17 (1.8–2.5)	2.39 (1–3)
Petiole width	0.2 (0.2–0.3)	0.31 (0.3–0.5)	0.4 (0.3–0.5)	0.23 (0.3–0.4)	0.3 (0.3–0.4)	0.3 (0.3–0.4)
Number of flowers per inflorescences	2	5* (2–8)	4* (2–6)	5 (2–8)	2 (2–4)	2 (2–4)
Bud length	1.2 (1–1.6)	1.47 (1.2–1.6)	1.48 (1.4–1.6)	1 (0.7–1.2)	1.5 (1.4–1.6)	1.65 (1.5–1.7)
Bud width	1 (0.9–1)	0.8 (0.8–1)	0.79 (0.7–0.9)	0.43 (0.3–0.6)	1 (0.8–1.1)	0.8 (0.7–0.8)
Bud length width ratio	1.2 (0.9–14)	1.81 1.69–2.23	1.87 (1.74–2.28)	2.39 (1.81–2.51)	1.68 (1.2–1.81)	2.06 (1.79–2.32)
Peduncle length	1 (0.8–1)	3.15* (1.5–6)	2.72* (2–3.5)	3.9 (2.5–5.5)	1.3 (1.2–1.5)	1.85 (1–2.5)
Peduncle width	0.5 (0.4–0.6)	0.3 (0.3–0.5)	0.4 (0.3–0.5)	0.2 (0.2–0.3)	0.5 (0.4–0.5)	0.4 (0.3–0.4)
Petal length	0.8 (0.7–1)	0.8 (0.8–1)	1 (0.9–1.1)	0.8 (0.7–0.9)	1.2 (1–1.2)	1 (0.9–1.1)
Petal width	0.2 (0.2–0.3	0.3 (0.3–0.4)	0.4 (0.3–0.4)	0.3 (0.2–0.4)	0.4 (0.3–0.4)	0.3 (0.2–0.3)
Stamen number	12 (9–14)	8*	4*	8	12 (8–16)	12 (8 – 16)
Stamen length	0.8 (0.8–1.1)	0.7 (0.7–0.9)	0.7 (0.5–0.7)	0.5 (0.4–0.6)	0.8 (0.4–1)	0.6 (0.4–0.8)
Style length	0.1 (0.06–0.12)	0.1 (0.08–0.12)	0.1 (0.08–0.12)	0.4 (0.3–0.5)	0.12 (0.08–0.15)	0.3 (0.28–0.41)

## Results

**Figure 1.**
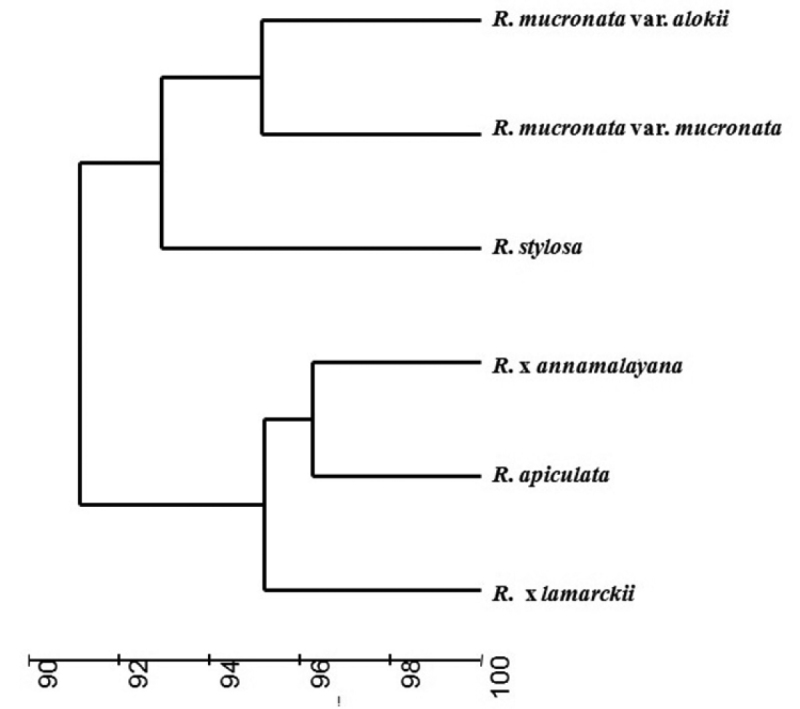
Cluster dendrogram (group average) showing similarity among the *Rhizophora* species of the ANI.

The morphometric analysis shows that Rhizophora mucronata var. alokii has closest similarity with *Rhizophora mucronata* than to other *Rhizophora* taxa (Fig. 1). However, attributes such as leaf length, length-width ratio, petiole length, peduncle length, number of flowers and stamen number are significantly different (p < 0.05) between the two taxa (Table 1).

## Taxonomic treatment

### Rhizophora mucronata var. alokii

Taxon classificationPlantaeMalpighialesRhizophoraceae

P.Ragavan var. nov.

urn:lsid:ipni.org:names:77148139-1

#### Material.

**Figure 2.**
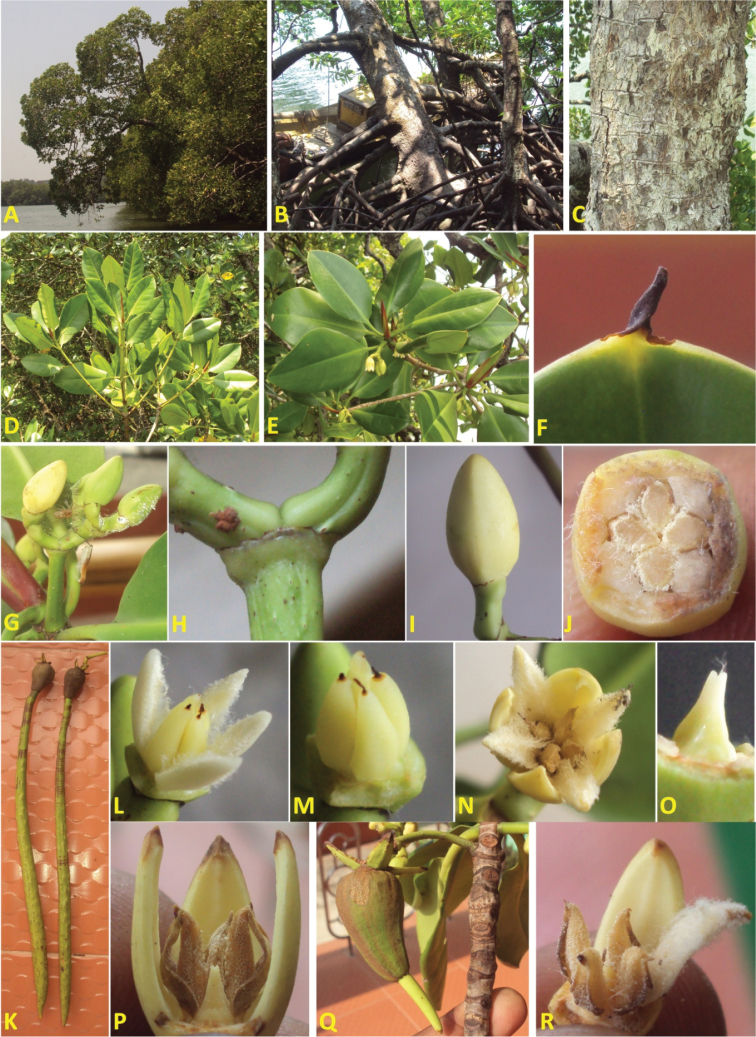
Rhizophora mucronata var. alokii (**A**) habit (**B**) stem base with stilt roots (**C**) bark (**D**) branches (**E**) leafy branch end with flowers (**F**)leaf apex with mucro (**G**) inflorescence (**H**) minute bract at dichotomous inflorescence branch (**I**) mature bud with minute bracteole below calyx (**J**) cross section of bud (**K**) mature propagules (**L**) thick leathery petal (**M**) stamens (**N**) flower (**O**) pistil showing four-sided ovary (**P**) flower with one petal removed (**Q**) pear-shaped fruit (**R**) stamens with pollen.

India. North Andaman: Austin Creek, mangrove forest (Fig. 2A), 12°52'36.9"N, 92°50'40.2"E, 3 April 2014, leg. P. Ragavan, PBL 31001 and 31002 (holotype: PBL).

*Tree*: columnar to spreading, height to 20 m, evergreen (Fig. 2A). *Bark*: dark brown, friable, fissured horizontally (Fig. 2C). *Roots*: both stilt roots and aerial roots growing from lower branches, stilt roots are highly conspicuous arching above ground to 2 m (Fig. 2B). *Leaves*: simple, opposite, green to dark green, elliptical to broadly elliptical (Fig. 2D, E), laterally folded, underside with numerous dark spots, 7–13 × 4–8.5 cm, length to width ratio averaging 1.69 (not greater than 1.8), apex obtuse with pointed mucro, 0.4–0.5 cm long (Fig. 2F), base cuneate, margin entire; petiole green, 1.5–3 × 0.3–0.5 cm. *Inflorescences*: axillary, 2–6 flowered (Fig. 2G); bract and bracteoles minute (Fig. 2H); peduncle 2–3.5 × 0.3–0.5 cm; pedicel stout; *Mature flower*: ellipsoidal, creamy white (Fig. 2I), 1.4–1.6 × 0.7–0.9 cm, length to width ratio *ca.* 1.87, cross section slightly four-sided (Fig. 2J); calyx lobes 4, thicker than *Rhizophora mucronata*, yellowish white, apex acute; petals 4, thick, leathery, folded laterally, creamy white, velvety and hairy on the margin (Fig. 2L, N), 0.9–1.1 × 0.3–0.4 cm; stamens 4, 0.5–0.7cm long (Fig. 2M, N); style bilobed (Fig. 2O), 0.8–0.12 cm long, seated on four sided domed ovary (Fig. 2P). *Mature fruits*: pear-shaped, brown, 4–5 × 2.5–3.5 cm, calyx persistent with erect lobes (Fig. 2Q). *Mature hypocotyls*: 40–60 cm long, green, tip pointed, 1.5–1.7 cm wide at widest point (Fig. 2K); plumule green, 2–3 cm long.

#### Distribution.

Rhizophora mucronata var. alokii is currently known only from Austin Creek, North Andaman, India.

#### Habitat and ecology.

It grows in a mangrove forest along the banks in an intermediate estuarine position in association with *Rhizophora apiculata*, *Rhizophora mucronata* and *Ceriops tagal*.

#### Phenology.

Flowering December to March; fruiting April to July.

#### Etymology.

Named in honour of Dr. Alok Saxena (Principal Chief Conservator of Forests) for his inspiration and his outstanding contribution to mangrove conservation in the ANI.

#### Conservation status.

Rhizophora mucronata var. alokii was collected only from Austin Creek (North Andaman Islands). At this site ca. 15 individuals were observed and hence it is assumed to be rare. At present, until further areas can be sampled the species can be accessed as “Data Deficient” (DD), using the criteria of IUCN (2001).

## Discussion

*Rhizophora* species are very similar and can be difficult to distinguish (Lo 2003). The key distinguishing characters of *Rhizophora* spp. in the ANI are given in Table 2. The identification of *Rhizophora apiculata* is not problematic because it differs from the other species within its range in many characters, including having apiculate leaves with spinose mucronate tips, bi-flowered inflorescences borne on short peduncles below the leaves, short styles and a swollen, corky, brown bract below the calyx. However, dark spots are present on the leaf undersides of *Rhizophora apiculata* from India to southeast Asia and northern Papuasia; they are absent in southern Papuasia and northern Australia (Duke et al. 2002). The number of calyx lobes varies geographically; throughout most of the species range there are four lobes but in Australia there are three to six lobes (Duke et al. 2002).

**Table 2.** Diagnostic characters of *Rhizophora* species of the ANI. The hybrids do not produce seeds so hypocotyl characters are not present in them and therefore not included in the table.

Component	Attributes	*Rhizophora apiculata*	Rhizophora mucronata var. mucronata	Rhizophora mucronata var. alokii	*Rhizophora stylosa*	Rhizophora × annamalayana	Rhizophora × lamarckii
Leaves	Leaf shape	narrowly elliptic	ovate, broader at base	elliptic	narrowly obovate broader at apex	broadly elliptic	narrowly elliptic
	Leaf apex	acute	acute	obtuse	obtuse	acute	acute
	Leaf base	cuneate	broadly acute to rounded	cuneate	cuneate	cuneate	attenuate to cuneate
Inflorescences	Position relative to leaves	matures below	matures within	matures within	matures within	matures within	mature within
	Flower number	2	2–8	2–6	2–8	2–4	2–4
	Juncture number	1	1 to 3	1 to 3	1 to 3	1 to 2	1 to 2
	Bract condition	corky	smooth, minute	smooth, minute	smooth, conspicuous	smooth, swollen	smooth swollen
Mature flower bud(closed)	Bud length	1–1.6 cm	1.2–1.6 cm	1.4–1.6 cm	0.7–1.2 cm	1.4–1.6 cm	1.5–1.7 cm
	Bud width	0.9–1 cm	0.8–1 cm	0.7–0.9 cm	0.3–0.6 cm	0.8–1.1 cm	0.7–0.8 cm
	Shape x-section	rounded	rounded	slightly four- sided	rounded	four-sided	rounded
	Bud length /width ratio	1.2	1.81	1.87	2.39	1.68	2.06
	Petal x-section	flat	enclose stamens	thick folded	enclose stamens	curved	curved
	Petal margin	glabrous	Hairy	velvety hairy	hairy	slightly hairy	slightly hairy
	Style length	0.08–0.12 cm	0.08–0.12 cm	0.08–0.1 cm	0.3–0.4 cm	0.08–0.12 cm	0.2–0.4 cm
	Stamen number	9 to 14	8	4	8	8–16 in two whorls	8–16 in one whorls
Mature hypocotyls	Expanded fruit	cork -like	pear- like	pear- like	pear-like		
	Hypocotyl length	20–40 cm	50–80 cm	40–60 cm	21–35 cm		
	Distal shape	bluntly pointed	narrowly pointed	narrowly pointed	narrowly pointed		

*Rhizophora* hybrids are recognized by intermediate morphology and absence of advanced reproductive stages (Tomlinson 1986). Both Rhizophora × lamarckii and Rhizophora × annamalayana are distinguished from *Rhizophora apiculata* by their smooth green bract and 2-4 flowered inflorescences within the leaf axils. Rhizophora × annamalayana is distinguished from Rhizophora × lamarckii by its broader leaves (length: width ratio <1.8 *vs* >1.8), and shorter style (<1.5 mm *vs.* > 1.5 mm) and stamens in two whorls *vs.* usually in one single whorl.

Distinguishing *Rhizophora mucronata* and *Rhizophora stylosa* is often problematic. Style length is the main feature used to differentiate these taxa; Ragavan et al. (2011) showed that in *Rhizophora mucronata* the style is short and the ovary elongate and tapering, similar to that in *Rhizophora apiculata*, whereas in *Rhizophora stylosa* the style is long and ovary is short, although intermediates are found. The two species also differ in that *Rhizophora stylosa* has prominent, two-lobed bracts and bracteoles, smaller buds, obovate leaves, smaller fruits and shorter propagules.

All previously described *Rhizophora* species have eight or more stamens, whereas Rhizophora mucronata var. alokii has four stamens. Rhizophora mucronata var. alokii closely resembles Rhizophora mucronata var. mucronata in its minute bract and bracteoles, bark texture, and bud shape, but can be distinguished not only by stamen number but also by its dense foliage, laterally folded leaves, thick leathery petals with dense hairs, shorter peduncle, and four-sided ovary. It can be difficult to distinguish var. alokii from var. mucronata without the presence of flowers. Differences in flowering time is likely to make this taxon reproductively isolated. A key to the ANI species of *Rhizophora* is given below.

### Key to Rhizophora spp. of ANI

**Table:** 

1	Peduncle shorter than petiole	**2**
–	Peduncle as long as or longer than petiole	**4**
2	Mature flower bud and fruits below the leaves; inflorescences two-flowered; bract corky, brown; hypocotyl present	***Rhizophora apiculata***
–	Mature flower buds within the leaves; inflorescences 2-4-flowered; bract smooth and green; hypocotyls not present	**3**
3	Leaves broadly elliptical; styles 0.8–1.2 mm long; stamens in two whorls, inner shorter; mature flower bud four-sided in cross-section	**Rhizophora × annamalayana**
–	Leaves narrowly elliptical; styles 2–3 mm long; stamens in one whorl; mature flower bud rounded in cross-section	**Rhizophora × lamarckii**
4	Stamens 4, petals thick and leathery, densely hairy	**Rhizophora mucronata var. alokii**
–	Stamens 8; petals thin, hairy at margin	**5**
5	Bract and bracteoles minute; style 1 mm long, seated on elongate, tapering ovary; hypocotyls 50–80 cm long	**Rhizophora mucronata var. mucronata**
–	Bract and bracteoles prominent, forming two-lobed, cup-like structure; style 3-4 mm, seated on short ovary; hypocotyls 20–40 cm long	***Rhizophora stylosa***

## Supplementary Material

XML Treatment for Rhizophora mucronata var. alokii

## References

[B1] Duke NC (2010) Overlap of eastern and western mangrove in the South western Pacific: hybridization of all three *Rhizophora* (Rhizophoraceae) combinations in New Caledonia. Blumea 55: 171–188. doi: 10.3767/000651910X527293

[B2] Duke NC Ball MC Ellison JC (1998) Factors influencing biodiversity and distributional gradients in mangroves. Global Ecol Biogeog Lett 7: 27–47. doi: 10.2307/2997695

[B3] Duke NC Bunt JS (1979) The genus *Rhizophora* (Rhizophoraceae) in northeastern Australia. Australian Journal of Botany 27: 657–678. doi: 10.1071/BT9790657

[B4] Duke NC Lo EYY Sun M (2002) Global distribution and genetic discontinuities of mangroves – emerging patterns in the evolution of *Rhizophora*. Trees 16: 65–79. doi: 10.1007/s00468-001-0141-7

[B5] FSI (2013) State of forest report. Forest Survey of India (FSI), Dehra Dun, 33–37.

[B6] IUCN (2001) IUCN Red List categories and criteria. Version 3.1, Prepared by the IUCN Species Survival Commission. IUCN, Gland, Switzerland, and Cambridge, United Kingdom, 32 pp.

[B7] Lo EYY (2003) Phylogenetic relationships and natural hybridization in the mangrove genus *Rhizophora* from the Indo-West Pacific region. M.Sc. Thesis, University of Hong Kong, Hong Kong, 198 pp.

[B8] Mandal RN Naskar KR (2008) Diversity and classification of Indian mangroves: a review. Tropical Ecology 49: 131–146.

[B9] Ng WL Chan HT Szmidt AE (2013) Molecular identification of natural mangrove hybrids of *Rhizophora* in Peninsular Malaysia. Tree Genet. Genomes 9: 1–10. doi: 10.1007/s11295-013-0619-7

[B10] Ragavan P Saxena M Coomar T Saxena A (2011) Preliminary study on natural hybrids of genus *Rhizophora* in India. ISME/ GLOMIS Electronic Journal 9: 13–19.

[B11] Tomlinson PB (1986) The botany of mangroves. Cambridge University Press, Cambridge, 413 pp.

